# A systematic review with meta-analysis: Traditional Chinese tuina therapy for insomnia

**DOI:** 10.3389/fnins.2022.1096003

**Published:** 2023-01-25

**Authors:** Zheng Wang, Hui Xu, Hang Zhou, Yang Lei, Lulu Yang, Juan Guo, Yuxia Wang, Yunfeng Zhou

**Affiliations:** ^1^College of Acupuncture and Massage, Henan University of Chinese Medicine, Zhengzhou, China; ^2^Tuina Department, The Third Affiliated Hospital of Henan University of Chinese Medicine, Zhengzhou, China; ^3^The First Affiliated Hospital of Henan University of Traditional Chinese Medicine, Zhengzhou, China

**Keywords:** tuina, insomnia, randomized controlled trial, meta-analysis, effective rate, Pittsburgh Sleep Quality Scale

## Abstract

**Background:**

With changes in the way of life and work, an increasing number of people are suffering from insomnia. In China, a traditional Chinese medicine method tuina is widely used for the treatment of insomnia. However, the evidence for tuina therapy for insomnia remains controversial. Therefore, this systematic review aimed to evaluate the effect of tuina therapy on the symptoms of patients with primary insomnia.

**Methods:**

From establishment to January 2022, a comprehensive literature search was conducted using seven electronic databases to identify randomized controlled trials of tuina therapy for insomnia. We used RevMan 5.4 software and the GRADEpro Guideline Development Tool to evaluate the quality of the included randomized controlled trials and perform the meta-analysis. The methodological quality of the included studies was assessed using the Cochrane risk-of-bias tool. Subgroup analysis was performed according to the different intervention methods. The I2 statistic was used to assess the heterogeneity.

**Results:**

Eighteen studies conducted from 2011 to 2021 were included, with a total of 1,471 patients. In terms of efficacy, tuina alone was superior to other treatments [odds ratio (OR), 3.46; 95% confidence interval (CI), 2.15, 5.55; *P* < 0.00001]; tuina combined with other treatments (acupuncture, scraping, auricular acupuncture, Suanzaoren decoction, estazolam) was more effective than other single therapies (OR, 3.99; 95% CI, 2.84, 5.61; *P* < 0.00001). In terms of Pittsburgh Sleep Quality Scale score, the improvement in insomnia patients by tuina alone was better than that of other treatments [standardized mean difference (SMD), −2.57; 95% CI, −2.98, −2.17; *P* < 0.00001], and tuina combined with other treatments (acupuncture, scraping, auricular point pressing, Suanzaoren decoction, estazolam) was better than other single therapies (SMD, −2.83; 95% CI, −2.98, −2.68; *P* < 0.00001).

**Conclusion:**

This meta-analysis revealed that tuina can significantly improve the clinical efficacy and sleep quality of patients with primary insomnia. This study provides a theoretical basis and treatment guidance for patients with primary insomnia.

**Systematic review registration:**

https://www.crd.york.ac.uk/prospero/, identifier CRD42022355742.

## 1. Introduction

Insomnia is a common sleep problem in modern society. It is also called “insomnia” or “blindness” in traditional Chinese medicine (TCM). It mainly manifests in a lack of sleep time and depth of sleep for various reasons. It may include dizziness, forgetfulness, fatigue, and other symptoms. Insomnia can be divided into two categories: primary and secondary. Primary insomnia refers to those who have insomnia symptoms despite lack of a clear cause or exclusion of what may be the cause of insomnia. The main causes of secondary insomnia include: (1) somatic organic diseases that affect the central nervous system; (2) alcohol, caffeine, or drugs that increase the excitability of the central nervous system; and (3) mental disorders, especially anxiety and depression, which are generally accompanied by insomnia. This review only focused on primary insomnia. Insomnia not only seriously affects the physical and mental health, and quality of life, of patients but also causes a variety of cardiovascular diseases (such as hypertension, coronary heart disease, and atherosclerosis), mental and psychological diseases (such as Alzheimer’s disease, anxiety, and depression), and is an important risk factor for cognitive impairment (Chen and Yuan, 2021). According to statistics, 45.5% of Chinese people have sleep problems, and the risk factors include age, sex, family history, genetic factors, and drugs. Age is an important risk factor for insomnia, and insomnia increases with age. The prevalence of insomnia in China has gradually increased (Zhang et al., 2012). TCM believes that the pathogenesis of insomnia always involves the decline of yang and yin, the loss of yin and yang, the disharmony of qi and blood, and the dysfunction of the viscera.

At present, Western medicine, such as benzodiazepines and antidepressants, is the main treatment for insomnia. However, long-term application of these medicines has certain side effects. For example, benzodiazepines are not only associated with drug dependence and tolerance, but also contribute to the incidence of Alzheimer’s disease, and some antidepressants may cause weight gain (Ye et al., 2017). TCM has a long history in treating insomnia, and it has been recorded in books such as “The Yellow Emperor’s Classic of Internal Medicine,” “On the Origin and Symptoms of Various Diseases,” and “Puji Fang.” Among them, tuina, as an important part of traditional medicine, has received attention in the treatment of insomnia owing to its advantages of simple administration, high safety, and good social and economic benefits. Tuina, a non-pharmacological intervention using fingers and strength, was developed from ancient therapeutic art. Tuina is a treatment based on TCM Zang-Fu organ and meridian theories, and integrates modern scientific knowledge (such as biomechanical function, anatomy, pathology, and physiology) with traditional practice. However, there is currently a lack of randomized controlled comparisons of the effectiveness of tuina compared with other intervention methods. This article uses a meta-analysis to comprehensively evaluate the effectiveness and safety of tuinas compared with other intervention methods to provide reliable evidence for clinical practice.

## 2. Methods

### 2.1. Trial registration

This systematic review was prospectively registered with the International Prospective Register of Systematic Reviews (number: CRD42022355742).

### 2.2. Search strategy

The following Chinese databases were searched: China National Knowledge Infrastructure, Wanfang, VIP, China Biomedical Literature Database; the international databases included PubMed, Embase, Cochrane Library, and Web of Science. The retrieval period was from the establishment of the database to August 2022. The search adopted the method of subject words + free words. Chinese search terms included: “insomnia,” “tuina,” “tuinas,” and “random control.” English search terms included: “insomnia,” “sleeplessness,” “agrypnia,” “tuina,” “massage,” “manipulation,” “randomized controlled trial,” and “RCT.”

### 2.3. Study selection

Literature screening was performed independently by two reviewers according to the inclusion and exclusion criteria. The inclusion criteria for this review were as follows. (1) Type of study: randomized controlled trial (RCT). (2) Participant type: Participants with a clinical diagnosis of insomnia, age, sex, course of disease, race, nationality, and TCM syndrome were not limited. (3) Types of intervention: The experimental group was treated with simple tuina therapy, while the control group was treated with treatments other than tuina therapy, such as acupuncture, moxibustion, and drugs. The experimental group received tuina + other therapy, and the control group received another therapy. There were no restrictions on the operation time, specific techniques, parts, acupoint selection, and treatment course of the tuina. (4) Outcome types: The total clinical response was the primary outcome, and the Pittsburgh Sleep Quality Index (PSQI) was the secondary outcome. We excluded (1) quasi-randomized RCT and non-randomized trials, (2) duplicate publications, and (3) studies without the full text available or missing data. Any disagreements were resolved through discussions between the two reviewers.

### 2.4. Data extraction

Two researchers independently searched the literature, performed preliminary screening according to the title and abstract, read the full text of the studies meeting the inclusion criteria, screened again according to the inclusion and exclusion criteria, and conducted a reference search for the included studies. Data extraction was performed for the included literature, including literature title, first author, publication year, sample size, intervention method, patient baseline data, outcome indicators, and the occurrence of adverse conditions. Any disagreement was discussed by all those involved in the audit.

### 2.5. Quality assessment

Two investigators evaluated the methodological quality of the included studies using the Cochrane risk bias assessment tool. According to the evaluation criteria, the following five items were rated as low, unclear, and high risk: ① generation of random allocation sequence; ② allocation concealment; ③ blinding of investigators and participants; ④ blind evaluation of study outcomes; and ⑤ completeness of outcome data. Any disagreements were resolved by obtaining consensus from all reviewers.

### 2.6. Data synthesis and analysis

First, a heterogeneity Q-test was performed. For results with *p* > 0.10, multiple similar studies were considered to be homogenous. If *p* > 0.10, and 0 ≤ *I*^2^ ≤ 50%, the combined analysis between the study results used a fixed effect model; if *p* ≤ 0.10, or *I*^2^ > 50%, the results of multiple similar studies were considered heterogeneous, and sensitivity analysis was performed first. A random effect model was used for combined analysis. Continuous variables used the standardized mean difference (SMD) with 95% confidence interval (CI), and dichotomous variables used the odds ratio (OR) with 95% CI to indicate the effect size; the test results were listed in a forest plot. Subgroup analysis was performed according to the different intervention methods. If more than 10 articles were included in the analysis, a funnel plot was drawn to analyze publication bias. Statistical significance was set at *p* < 0.05. Statistical analysis and the meta-analysis were performed using Review Manager version 5.4 (The Cochrane Collaboration, London, England).

## 3. Results

### 3.1. Search and selection

**FIGURE 1:**
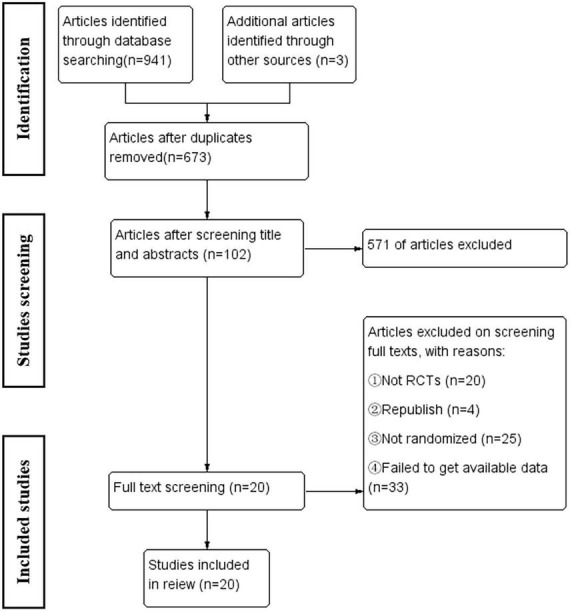
Flow chart for the review.

A total of 941 related articles were obtained, and 673 articles remained after the software was deduplicated. According to the inclusion and exclusion criteria, 20 articles were finally included. The inclusion process is illustrated in Figure 1.

### 3.2. Characteristics of the included studies

The included studies (Ye et al., 2017) were all Chinese RCTs, with a total of 1,713 insomnia patients, 858 in the treatment group and 855 in the control group. Ten studies used tuina alone to treat insomnia. The intervention methods in the control group included estazolam, acupuncture, Longdan Xiegan pills, Jieyu Anshen granules, Guipi decoction, and auricular point sticking. Eight studies used tuinas in combination with other therapies to treat insomnia. The control group received acupuncture, scraping, estazolam, Suanzaoren decoction, and auricular acupuncture. The course of tuina therapy was 14–30 days, and the majority were 20 days. Each tuina treatment time was 20–40 min, and the frequency was mostly once per day. Primary outcomes included the overall response rate and PSQI. The basic characteristics of the included studies are shown in Table 1.

**TABLE 1:** Randomized controlled trials evaluating the effects of tuina for insomnia.

References	Sample size	Duration (year)	Follow-up (months)	Experimental group intervention	Control group intervention	Main outcomes
Feng, 2017	100 100	3.1 ± 0.5 3.2 ± 0.8	/	Acupuncture (30 min, 1/day, 21 days)	Tuina + Acupuncture (1/day, 21 days)	Overall efficiency, PSQI
Liu, 2019	30 30	/	/	Longdan Xiegan Pills (2/day, 10 days)	Tuina (30 min, 1/day, 10 days)	Overall efficiency, PSQI
Luo, 2012	30 30	1.21 ± 0.52 1.19 ± 0.56	/	Acupuncture (20 min, 1/day, 15 days)	Tuina (30 min, 1/day, 15 days)	Overall efficiency, PSQI
Pan, 2018	29 28	/	/	Estazolam (1/day, 14 days)	Tuina (1/day, 14 days)	Overall efficiency, PSQI
Pang et al., 2015	77 73	2.25 ± 1.31 2.42 ± 1.15	/	Estazolam (1/day, 30 days)	Tuina (20 min, 1/day, 30 days)	Overall efficiency, PSQI
Shi et al., 2017	45 42	4.78 ± 4.56 4.56 ± 4.23	/	Acupuncture (20 min, 1/day, 20 days)	Tuina + Acupuncture (1/day, 20 days)	Overall efficiency, PSQI
Tang et al., 2015	38 38	1.75 ± 1.47 1.67 ± 1.30	/	Estazolam (1/day, 30 days)	Tuina (1/day, 30 days)	Overall efficiency, PSQI
Wang, 2019	40 40	3.4 ± 1.1 3.3 ± 0.8	/	Acupuncture (30 min, 1/day, 21 days)	Tuina + Acupuncture (1/day, 21 days)	Overall efficiency, PSQI
Wang, 2020	44 44	/	/	Scraping (1/day, 20 days)	Tuina + Scraping (1/day, 20 days)	Overall efficiency, PSQI
Wang, 2021	30 30	2.93 ± 0.79 2.98 ± 0.76	/	Acupuncture (30 min, 1/day, 30 days)	Tuina + Acupuncture (1/day, 21 days)	Overall efficiency, PSQI
Wei et al., 2013	40 40	2.18 ± 1.15 2.2 ± 1.12	/	Estazolam (1/day, 20 days)	Tuina (1/day, 20 days)	Overall efficiency, PSQI
Wu, 2019	30 30	/	/	Guipi Tang (1/day, 4 weeks)	Tuina (25 min, 3/week, 4 weeks)	Overall efficiency, PSQI
Xu, 2012	30 30	1.43 ± 1.07 1.59 ± 1.38	/	Auricular pressure (1/3 days, 30 days)	Tuina (30 min, 1/day, 30 days)	Overall efficiency, PSQI
Yu, 2013	28 31	/	/	Guipi Tang (3/day, 28 days)	Tuina (30 min, 1/day, 14 days)	Overall efficiency, PSQI
Zhang et al., 2011	30 36	/	/	Jieyu Anshen Granules (2/day, 21 days)	Tuina (1/day, 15 days)	Overall efficiency, PSQI
Zhang et al., 2020	30 30	2.4 ± 1.2 1.8 ± 0.9	/	Estazolam (1/day, 20 days)	Tuina (20 min, 1/2 days, 20 days)	Overall efficiency, PSQI
Zhang, 2021	35 35	0.56 ± 0.28 0.55 ± 0.29	/	Auricular pressure (1/2days, 20 days)	Tuina + Auricular pressure (1/day, 18 days)	Overall efficiency, PSQI
Zhou et al., 2007	84 82	/	/	Guipi Pills (3/day, 15 days)	Tuina (3/day, 15 days)	Overall efficiency, PSQI
Zhou et al., 2017	37 37	3.6 ± 2.1 3.7 ± 2.2	/	Suanzaoren Tang (2/day, 20 days)	Tuina + Suanzaoren Tang (1/day, 20 days)	Overall efficiency, PSQI
Zhuang, 2020	50 50	4.04 ± 0.57 4.03 ± 0.56	/	Estazolam (1/day, 20 days)	Tuina + Estazolam (1/day, 20 days)	Overall efficiency, PSQI

PSQI, Pittsburgh Sleep Quality Index.

### 3.3. Methodological quality

**FIGURE 2:**
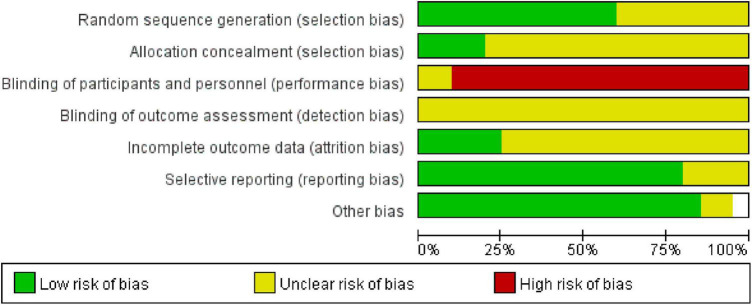
Risk of bias in included studies.

**FIGURE 3:**
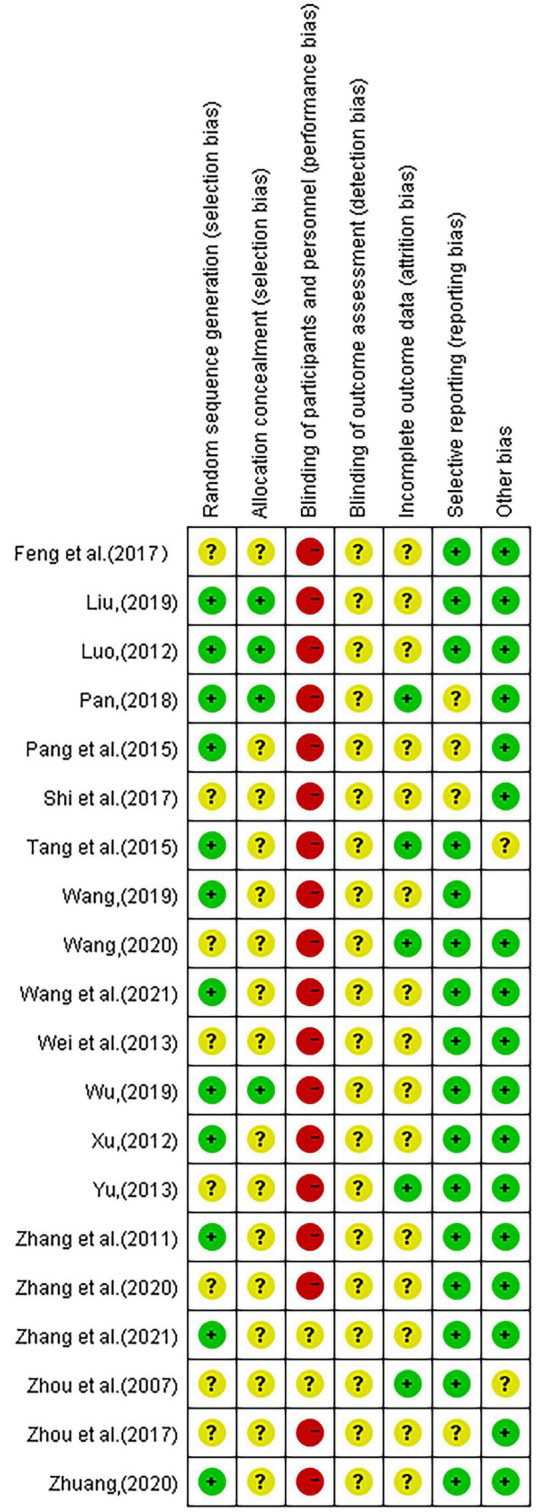
Risk of bias in included studies.

The 20 included studies applied the principle of randomization and most of the studies had good methodological quality. Among them, eight (Zhang et al., 2011; Luo, 2012; Xu, 2012; Pang et al., 2015; Wu, 2019; Wang, 2019, 2020; Zhuang, 2020) studies used the random number table method, two (Pan, 2018; Zhang et al., 2020) studies were randomly assigned according to the order of visits, one (Liu, 2019) study used the envelope random method, and one ^[4]^ study used computer-generated random numbers. Other studies did not describe the randomized methods used. Only four (Luo, 2012; Pan, 2018; Liu, 2019; Wu, 2019) studies had detailed hidden assignments. All of the included RCTs were free of subject blinding and treatment. One study (Yu, 2013) described the reasons for dropout in detail, and another study (Pan, 2018) only mentioned the number of people who were compared for efficacy or the intervention effect, and the report with an incomplete outcome was judged as “low risk.” In three studies (Pang et al., 2015; Shi et al., 2017; Pan, 2018), although cases were dropped, the reasons were not described, and it was not possible to determine whether they were selective outcome reports. No other biases were evident in the included studies. See Figures 2, 3.

### 3.4. Synthesis of the results

#### 3.4.1. Overall efficacy

**FIGURE 4:**
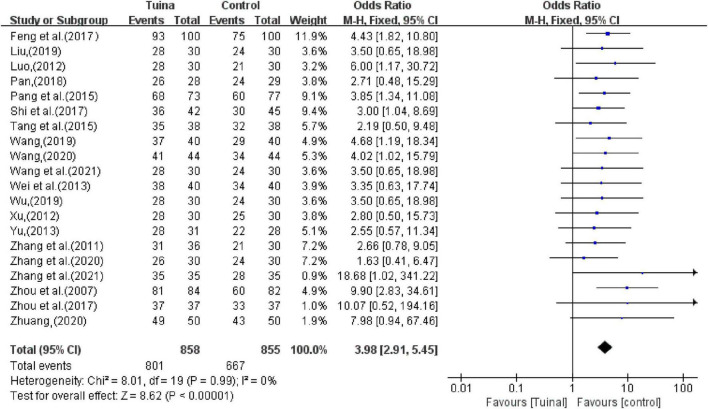
Meta-analysis of objective response rate (ORR).

In terms of the total effective rate, 20 studies reported that tuina can significantly improve the clinical symptoms of insomnia patients better than the control group (OR, 3.98; 95% CI, 2.91, 5.45; *P* < 0.00001, *I*^2^ < 50%, Figure 4). The sensitivity analysis showed that the above results were similar and not significantly different.

**FIGURE 5:**
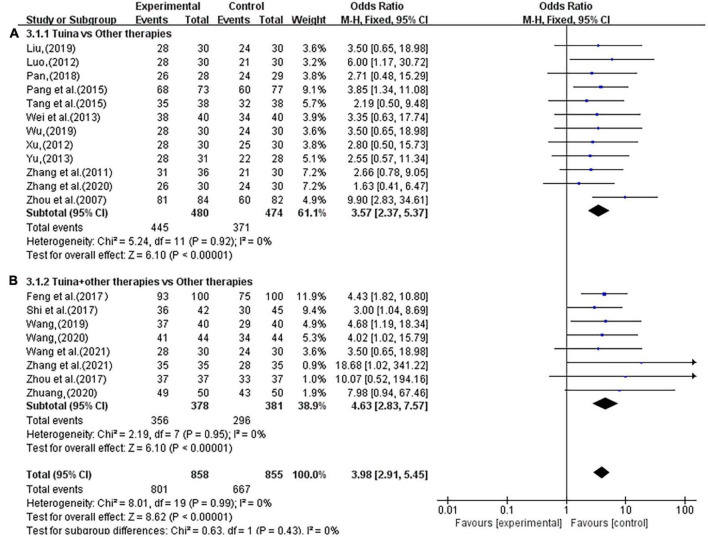
Forest plot of tuina for objective response rate (ORR). **(A)** Tuina vs. Other therapies. **(B)** Tuina + Other therapies vs. Other therapies.

In the subgroup analysis, as an independent treatment, tuina therapy improved insomnia better than the other treatments (OR, 3.57; 95% CI, 2.37, 5.37; *P* < 0.00001, Figure 5A). In studies of tuina combined with other therapies, the efficacy was better than that of other therapies alone (OR, 4.63; 95% CI, 2.83,7.57; *P* < 0.00001; Figure 5B). The sensitivity analysis showed that the above results were similar and not significantly different.

**FIGURE 6:**
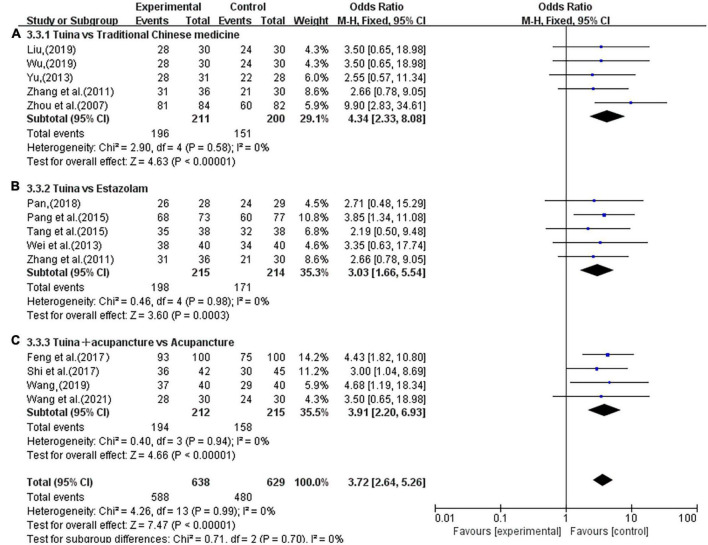
Subgroup analysis of tuina and different interventions in ORR. **(A)** Traditional Chinese Medicine (TCM). **(B)** Estazolam. **(C)** Acupuncture.

The subgroup analysis also showed that the use of tuina alone improved clinical symptoms in insomnia patients better than herbal treatment alone (OR, 4.34; 95% CI, 2.33, 8.08; *P* < 0.00001, Figure 6A) and eszopiclone treatment alone (OR, 3.03, 95% CI, 1.66,5.54; *P* = 0.0003; Figure 6B). Among the combination therapies, tuina combined with acupuncture was superior to acupuncture alone (OR, 3.91, 95% CI, 2.20,6.93; *P* < 0.00001; Figure 6C). The sensitivity analysis yielded similar and non-significant differences in these results.

#### 3.4.2. PSQI

**FIGURE 7:**
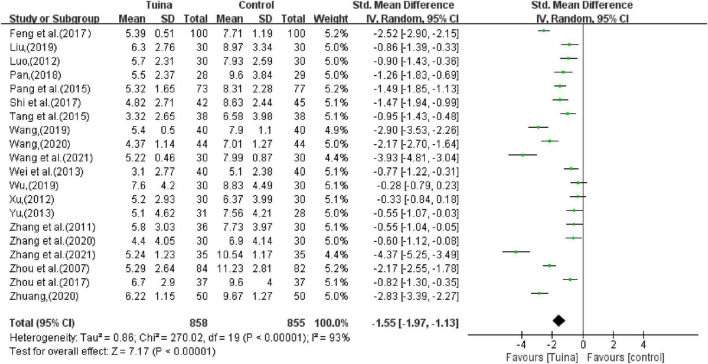
Meta-analysis of Pittsburgh Sleep Quality Index (PSQI).

The combined results of the PSQI score showed that tuina therapy could improve the sleep status of patients with insomnia (SMD, −1.55; 95% CI, −1.97, −1.13; *P* < 0.00001; *I*^2^ > 50%, Figure 7). The sensitivity analysis showed that the above results were similar and not significantly different.

**FIGURE 8:**
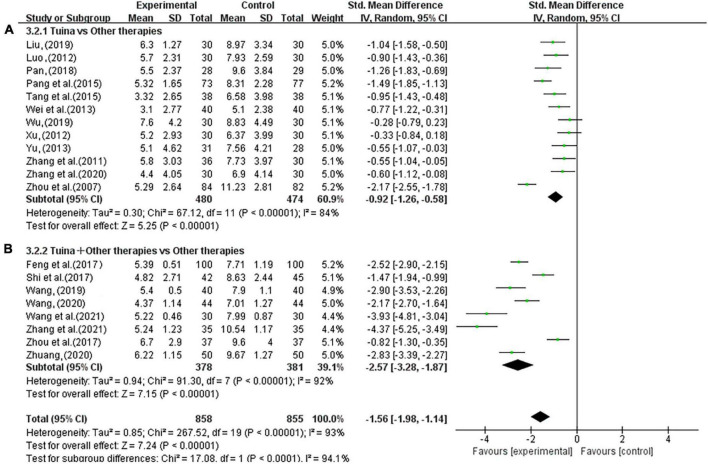
Forest plot of tuina for Pittsburgh Sleep Quality Index (PSQI). **(A)** Tuina vs. Other therapies. **(B)** Tuina + Other therapies vs. Other therapies.

In the subgroup analysis, tuina alone was lower than other treatments in the total PSQI score and could better improve the PSQI score of insomnia patients (SMD, −0.92; 95% CI, −1.26, −0.58; *P* < 0.00001, Figure 8A). Tuina combined with other therapies in the treatment of insomnia could significantly improve the PSQI score of patients compared to other therapies alone (SMD, −2.57; 95% CI, −3.28, −1.87; *P* < 0.00001, Figure 8B). The sensitivity analysis showed that the above results were similar and not significantly different.

**FIGURE 9:**
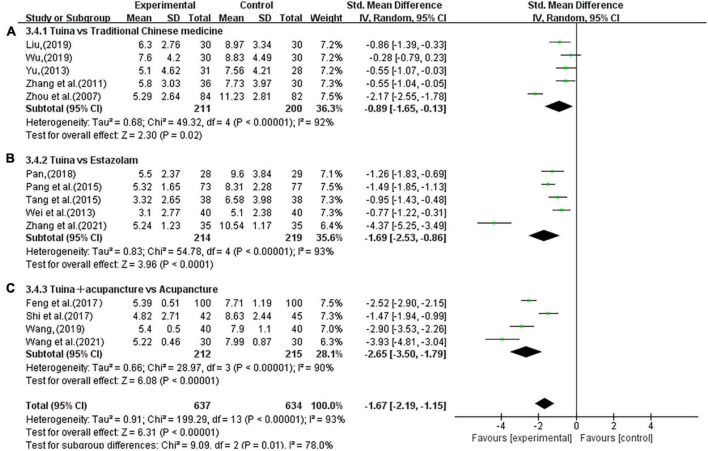
Subgroup analysis of tuina and different interventions in Pittsburgh Sleep Quality Index (PSQI). **(A)** Traditional Chinese Medicine (TCM). **(B)** Estazolam. **(C)** Acupuncture.

The subgroup analysis showed that the use of tuina alone improved the PSQI scores of insomnia patients better than herbal treatment alone (SMD; −0.89; 95% CI, −1.65, −0.13; *P* < 0.00001, Figure 9A) and eszopiclone treatment alone (SMD, −1.69; 95% CI, −2.53, −0.86; *P* < 0.00001, Figure 9B). Among the combination therapies, tuina combined with acupuncture was more effective than acupuncture alone (SMD, −2.65; 95% CI, −3.50, −1.79; *P* < 0.00001; Figure 9C). The sensitivity analysis yielded similar and non-significant differences in these results.

#### 3.4.3. Publication bias

**FIGURE 10:**
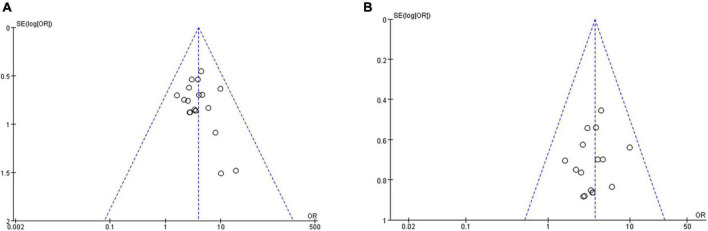
The funnel plots for tuina for primary insomnia. **(A)** Before sensitivity analysis. **(B)** After sensitivity analysis.

**FIGURE 11:**
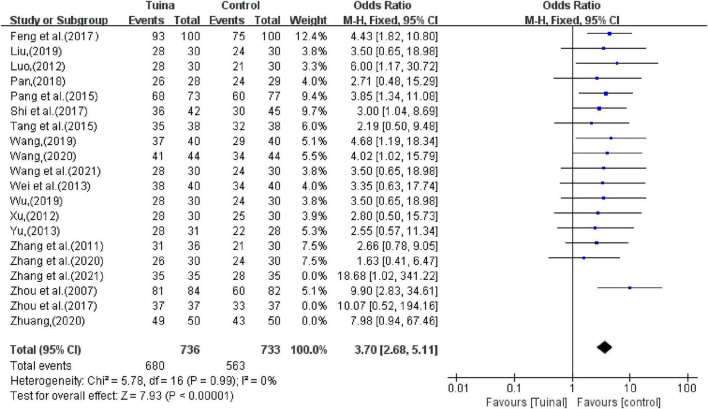
Forest plot of tuina for objective response rate (ORR) after sensitivity analysis.

The funnel plot of tuina in improving patients with primary insomnia included 20 RCTs (Figure 10A). The funnel plot was somewhat biased because the blobs were asymmetrical. Sensitivity analysis was carried out by excluding literature one by one, and it was found that the bias after excluding three studies (Zhou et al., 2017; Zhuang, 2020; Zhang, 2021) was low, and the spots were basically symmetrical, as shown in Figure 10B. The comprehensive analysis results after exclusion were relatively stable (OR, 3.70; 95% CI, 2.68,5.11; *P* < 0.00001; Figure 11). Reevaluation of the three studies revealed that the source of heterogeneity was possibly related to intervention frequencies, inconsistent times, and different indicator measures among institutions.

#### 3.4.4. Quality of evidence

The GRADEpro Guideline Development Tool was employed to assess the quality of all the outcomes from five aspects, including the risk of bias, inconsistency, indirectness, imprecision, and other considerations. The included studies have some defects in randomization, allocation concealment, and blinding, which were downgraded one level because of risk of bias. One outcome was downgraded one level in terms of consistency due to the high heterogeneity of the results. The final results revealed that five outcomes were of medium quality and six were of low quality, as shown in Table 2.

**TABLE 2:** GRADE evidence profile.

Outcomes (Trials)	Quality assessment					Group		Clinical efficacy and safety		Quality
	Risk of bias	Inconsistency	Indirectness	Imprecision	Publication bias	Experiment	Control	Relative ratio (95% CI)	Absolute (95% CI)	
ORR (20)	Serious^1^	No	No	No	Strongly suspected^2^	801/858 (93.1%)	667/855 (78.5%)	3.98 (2.91 to 5.45)	154 more per 1,000 (from 132 more to 171 more)	● ⊕⊕ ◯◯ low
PSQI (20)	Serious^2^	No	No	No	None	858	855	No	1.55 (1.97 to 1.13)	⊕⊕⊕◯ Moderate

ORR, objective response rate; PSQI, Pittsburgh Sleep Quality Scale.

^1^The included studies have certain defects in randomization, allocation concealment and blinding.

^2^There is evidence of publication bias, Egger’s test, *p* < 0.05.

#### 3.4.5. Adverse events

None of the included studies reported whether there were adverse events or not.

## 4. Discussion

This systematic review and meta-analysis evaluated the efficacy of tuinas in the treatment of primary insomnia. The results show that tuina therapy can significantly improve the clinical symptoms and PSQI scores of patients with primary insomnia. The curative effect of pure tuina in treating insomnia is better than that of acupuncture, estazolam, TCM, and other treatments, and the curative effect of tuina combined with acupuncture, Suanzaoren decoction, estazolam, and other treatments is better than that of acupuncture alone. However, there is not enough evidence to support that tuina alone is superior to acupuncture and auricular sticking alone in improving the efficacy in insomnia patients. There is also insufficient evidence to prove the efficacy of tuina combined with estazolam and TCM in the treatment of insomnia, which is better than that of estazolam and TCM alone. Moreover, there is insufficient evidence on the long-term efficacy of tuina in insomnia patients, which may be related to the lack of included studies and the lack of follow-up in most studies.

In the included studies, other combined therapies included acupuncture, TCM, estazolam, scraping, and auricular sticking; however, due to the limited number of included studies, only subgroup analyses were performed on studies of tuina combined with acupuncture. Furthermore, the effect of tuina therapy on the mental health of patients with primary insomnia has not been addressed in any study. We found that tuina therapy has a certain effect on improving anxiety and depression in patients with insomnia (Luo, 2012; Pan, 2018; Wu, 2019). Tuina manipulation may act on the body surface and produce a stimulating effect by touching the tactile receptors on the skin, causing the excitation of tactile receptors, baroreceptors, and deep tissue pulling receptors, forming action potentials of different frequencies and numbers, and then through the complex ascending pathway of afferent nerves, reaching different nerve centers (Fang and Fang, 2013). Finally, the nerve-endocrine-endocrine pathway, one of the three intermediary pathways of the immune system, plays an extensive and effective role in regulation and treatment (Guo, 2009). In these three studies (Luo, 2012; Pan, 2018; Wu, 2019), the intervention methods of the experimental group were tuinas alone, and the combined therapy of tuinas was not included. Therefore, our review provides strong evidence for tuina therapy in the improvement of anxiety and depression in patients with primary insomnia.

In China, tuina is often used as adjunct therapy for neck pain. Our review included studies that used tuina plus acupuncture, herbal remedies, or medications to treat insomnia. Therefore, we reviewed the complementary and independent effects of tuina in the treatment of insomnia and conducted a subgroup analysis in the meta-analysis according to whether tuina was combined with other treatments. A previous systematic review (Yang et al., 2019) was consistent with the results of our review. Therefore, there is strong evidence in our review that tuinas can effectively treat insomnia.

Progress has also been made in the study of tuina for the treatment of insomnia by modulating neural circuitry. Current studies show that the anterior cingulate, amygdala, hippocampus, thalamus, and nucleus accumbens are involved in sleep-wake regulation in humans (Liu et al., 2020). When the body is in a state of arousal or when the ability to fall asleep is impaired, studies have identified associations between cognitive functions and dysregulation of the loops located in the limbic cortical system, which is closely associated with the formation of insomnia (Perrier et al., 2015). Gamma-aminobutyric acidergic neurons within the ventral lateral preoptic area of the hypothalamus (ventrolateral preoptic area) are considered to be the sleep nuclei, while neurons within the nuclei such as the nucleus “blue spot” and nodal papillary nuclei are referred to as the nuclei of arousal (Yi and Zhang, 2019). Many multifunctional nuclei are present in the lateral hypothalamic area involved in the sleep-wake cycle, eating, and metabolism (Stuber and Wise, 2016), and dysfunction of these nuclei can trigger insomnia symptoms. In a further study of brain mechanisms, the hypothalamic paraventricular nucleus-paraventricular nucleus neural circuit was identified as necessary for sleep-wake regulation (Liu, 2021). Researchers (Zhi et al., 2022) found that abdominal thrusting in an insomnia rat model significantly improved the neurotransmitters 5-HT and β-EP in the hypothalamus. They hypothesized that, through the observation of electroencephalogram activity, the information generated by abdominal thrusting could be transmitted to the hypothalamus through the lower centers of the brain to regulate secretion of the related neurotransmitters by altering the functional changes of sleep-wake nuclei, such as the nucleus blue spot and nodal papillary nucleus within the hypothalamus, thereby regulating excessive arousal and improving insomnia. Zhang (2022) used the vibration abdominal ring kneading method to intervene in PI model rats, and the results showed that the behavioral scores of rats improved while the number of corticotropin-releasing hormone receptors 1 and 2 and gamma-aminobutyric acid levels decreased in the hypothalamus after the tuina intervention. These findings suggest the key role of regulating the hypothalamic corticotropin-releasing hormone-receptor pathway in the treatment of insomnia using the vibration abdominal ring kneading method. Another study found (Hu, 2018) that the occipitofrontal neural circuit had an influential role in the process of tuina treatment in PI patients, with a positive correlation to improved sleep indicators.

Research has shown that the main causes of insomnia are hypothalamic-pituitary-adrenal axis dysfunction, changes in vagal tone, and central neurotransmitter disorders (Cheng et al., 2016). Tuina, as a non-drug therapy for insomnia, may exert pressure through tactile receptors, improve blood circulation, regulate the excitation and inhibition of the central system and cerebral cortex, increase the level of serotonin in patients with insomnia, and improve health. Sleep status (Yao, 2014), may be related to the improvement in patients’ clinical symptoms and PSQI scores. Tuina manipulation is gentle and deep, which can effectively relieve pressure on patients and relax skeletal muscles. Simultaneously, tuina manipulation can further regulate the central nervous system by stimulating the meridians and acupoints. This may be the potential mechanism by which tuina improves the anxiety and depression scores of patients with insomnia. However, its specific mechanism of action remains unclear.

According to the theory of TCM, the secretion of yang-ping is an important condition for the normal realization of the function of the viscera, and TCM believes that the loss of communication between yin and yang is the basic pathogenesis of insomnia. Under the influence of a variety of pathogenic factors, yang qi in the body does not meet with yin, and yang is prosperous and yin is weakened, resulting in dysfunction of the viscera and meridians, and sleep disturbance. Tuina acts on the meridians and acupoints of the human body through different manipulations, which can regulate blood circulation, balance yin and yang, accelerate blood circulation, promote the recovery of blood vessel walls, dilate capillaries, rebuild capillary networks, reduce blood viscosity, and improve cardiac function and blood pressure. This slows down gastrointestinal motility, promotes the secretion of digestive juice, regulates qi and blood, and balances yin and yang to achieve the purpose of treatment (Fan, 2015). According to the theory of TCM, the secretion of yang-ping is an important condition for the normal realization of the function of the viscera, and TCM believes that the loss of communication between yin and yang is the basic pathogenesis of insomnia. Under the influence of a variety of pathogenic factors, yang qi in the body does not meet with yin, and yang is prosperous and yin is weakened, resulting in dysfunction of the viscera and meridians, and sleep disturbance. Tuina acts on the meridians and acupoints of the human body through different manipulations, which can regulate blood circulation, balance yin and yang, accelerate blood circulation, promote the recovery of blood vessel walls, dilate capillaries, rebuild capillary networks, reduce blood viscosity, and improve cardiac function and blood pressure. This slows down gastrointestinal motility, promotes the secretion of digestive juice, regulates qi and blood, and balances yin and yang to achieve the purpose of treatment (Fan, 2015).

### 4.1. Limitations

This review had several limitations. First, only a few of the included studies mentioned the long-term efficacy, and the follow-up time did not exceed 6 months; therefore, there is still no direct and favorable evidence for the long-term efficacy of tuina in the treatment of insomnia. Second, the studies included were insufficient in terms of methodology and sample size, with large inconsistencies and precision, which had an impact on the outcome indicators; that is, the quality of evidence on tuina therapy for insomnia was low. Third, our review only included RCTs, but it is difficult to blind patients and therapists in tuina studies, which directly affects the quality of the final body of evidence. Implementing opaque outcome raters and implicit assignments could partially remedy these deficiencies, but only four studies used implicit assignments and two studies blinded outcome reviews. Fourth, the results may be influenced by the TCM syndrome types of insomnia, but subgroup analyses could not be performed due to the small number of included studies. Fifth, the results may be influenced by the genre, frequency, duration, and course of treatment of the different tuina manipulations. If there are enough eligible studies in the future, further review should focus on these parameters of tuina. Sixth, the results of the included studies were all positive, and there is a possibility of omission of negative results, which has an impact on publication bias. Finally, the included studies were all published in Chinese, suggesting that tuina therapy has limited use outside of China, hence more research on tuina therapy for insomnia is necessary.

## 5. Conclusion

Altogether, tuina can effectively improve the clinical efficacy and sleep quality of patients with primary insomnia, which is worthy of further promotion and application in clinical practice. However, considering the poor quality of the included studies, we need to carry out large-scale, multi center, high-quality rct to further confirm the results of this study.

## Data availability statement

The original contributions presented in this study are included in the article/supplementary material, further inquiries can be directed to the corresponding authors.

## Author contributions

ZW, HX, YW, and YZ designed the study and drafted the manuscript. HZ and LY performed literature searches. ZW and HZ identified and selected studies. LY and YL assessed methodological quality and extracted the data. ZW and HX performed data synthesis and analysis, respectively. ZW, HX, HZ, LY, and JG wrote the manuscript. All authors contributed to the manuscript and approved the submitted version.
